# Supporting Self-Care for Families of Children With Eczema With a Web-Based Intervention Plus Health Care Professional Support: Pilot Randomized Controlled Trial

**DOI:** 10.2196/jmir.3035

**Published:** 2014-03-04

**Authors:** Miriam Santer, Ingrid Muller, Lucy Yardley, Hana Burgess, Hannah Selinger, Beth L Stuart, Paul Little

**Affiliations:** ^1^Primary Care and Population SciencesUniversity of SouthamptonSouthamptonUnited Kingdom; ^2^Academic Unit of PsychologyFaculty of Social and Human SciencesUniversity of SouthamptonSouthamptonUnited Kingdom; ^3^Faculty of MedicineUniversity of SouthamptonSouthamptonUnited Kingdom

**Keywords:** child, primary care, randomized controlled trial, eczema, Internet, self-care

## Abstract

**Background:**

Childhood eczema, or childhood atopic dermatitis, causes significant distress to children and their families through sleep disturbance and itch. The main cause of treatment failure is nonuse of prescribed treatments.

**Objective:**

The objective of this study was to develop and test a Web-based intervention to support families of children with eczema, and to explore whether support from a health care professional (HCP) is necessary to engage participants with the intervention.

**Methods:**

We followed the PRECEDE-PROCEED model: regular emollient use was the target behavior we were seeking to promote and we identified potential techniques to influence this. LifeGuide software was used to write the intervention website. Carers of children with eczema were invited through primary care mail-out and randomized to 3 groups: (1) website only, (2) website plus HCP support, or (3) usual care. Patient-Oriented Eczema Measure (POEM) scores were measured online by carer report at baseline and at 12 weeks. Qualitative interviews were carried out with 13 HCPs (primarily practice nurses) and 26 participants to explore their experiences of taking part in the study.

**Results:**

A total of 143 carers were recruited through 31 practices. We found a decrease of ≥2 in follow-up compared with baseline POEM score in 23 of 42 (55%) participants in the website only group, 16 of 49 (33%) in the usual care group, and 18 of 47 (38%) in the website plus HCP group. Website use data showed that 75 of 93 (81%) participants allocated to the website groups completed the core modules, but less than half used other key components (videos: 35%; regular text reminders: 39%). There were no consistent differences in website use between the website only or the website plus HCP groups. Qualitative feedback showed that most HCPs had initial concerns about providing support for eczema self-care because this was not a condition that they felt expert in. However, HCPs reported productive consultations and that they found it helpful to use the website in consultations, while observing that some participants seemed to need more support than others. Qualitative interviews with participants suggested that HCP support was valued highly only by a minority, generally those who were less confident in their management of eczema or less confident using the Internet.

**Conclusions:**

Our pilot trial demonstrated the potential for greater improvements in POEM scores in both website intervention groups and that a full-scale trial is feasible. Such a trial would quantify the effectiveness and cost-effectiveness of this intervention to determine whether it should be widely promoted to families of children with newly diagnosed eczema. In this study population, HCP support was not strongly valued by participants and did not lead to better outcomes or website use than use of the Web-based intervention alone.

**Trial Registration:**

International Standard Randomized Controlled Trial Number (ISRCTN): 98560867; http://www.controlled-trials.com/ISRCTN98560867 (Archived by WebCite at http://www.webcitation.org/6NcxvMtgN).

## Introduction

Childhood eczema, or childhood atopic dermatitis, is very common, affecting more than 20% of children aged 5 years or younger at some point [1]. It can cause significant distress to the child and family because of sleep disturbance and itch [2]. The main cause of treatment failure is carers not using treatments correctly due to not understanding treatments, child refusal, or the therapy being too time-consuming [3]. Carers may need support in dealing with behavioral issues, such as scratching and sleep disturbance or children refusing topical treatments.

A Cochrane review found that most studies of parental education for childhood eczema have been small or of poor quality [4]. The National Institute for Health and Care Excellence (NICE) guideline on eczema in children [5] concluded that lack of education about therapy leads to poor adherence (ie, carers not using creams) and consequently to treatment failure.

Families of children with eczema express frustration that they do not receive enough support and information about how to manage the condition [6,7]. An Internet-delivered self-management intervention for carers offering information and support about childhood eczema to carers could address this gap. Web-based interventions have been shown to produce small but significant changes in health-related behaviors, particularly where intervention development is based on theory and multiple behavior techniques are incorporated [8]. In terms of patient outcomes, Web-based interventions for diabetes have shown small beneficial effects on blood glucose [9]. Among children and adults referred to secondary care for eczema, an eHealth package (including Internet-guided self-management and Web-based consultations) was found to be just as effective as usual face-to-face dermatology care, but was more cost-effective [10]. Internet-based self-management interventions have been found to lead to improved outcomes in other pediatric long-term illnesses, particularly asthma [11,12].

The effectiveness of computerized mental health interventions have been shown to be enhanced by health care professional (HCP) support or other social support [13]. A systematic review that included interventions aimed at lifestyle change, mental health, or chronic conditions suggested that increased human support is associated with increased adherence to interventions [14]. However, many of the interventions studied in chronic conditions involved clinic-based HCP support for conditions such as diabetes, arthritis, or chronic pain. It is not clear for which conditions and to what extent HCP support is necessary to engage users with Web-based interventions for chronic illness.

We developed a Web-based intervention aimed at improving management of childhood eczema by parents/carers. We sought to test the intervention in a pilot trial to investigate feasibility of intervention delivery and to gather preliminary information for a full-scale randomized controlled trial (RCT). We included 3 groups: (1) Web-based intervention plus usual care, (2) Web-based intervention plus HCP support plus usual care, and (3) usual care alone.

## Methods

### Development of Web-Based Intervention

The home page on the SPaCE (Supporting Parents and Carers of Children with Eczema) website had a link to allow users to access brief information about members of the research team (including University affiliations) and the medical experts who developed the website. Participants then followed 2 short compulsory modules (approximately 20 minutes) on “What is eczema?” and “Emollient moisturizers” before reaching a menu of modules reflecting common concerns of carers of children with eczema. These were identified through previous qualitative interviews and discussion with patient support groups.

We followed the PRECEDE-PROCEED model [15], using a team approach to identify relevant behaviors amenable to change and potential techniques for addressing these. Details of intervention development are presented in Textbox 1 and an overview of the intervention is given in Textbox 2.

**Figure 1:**
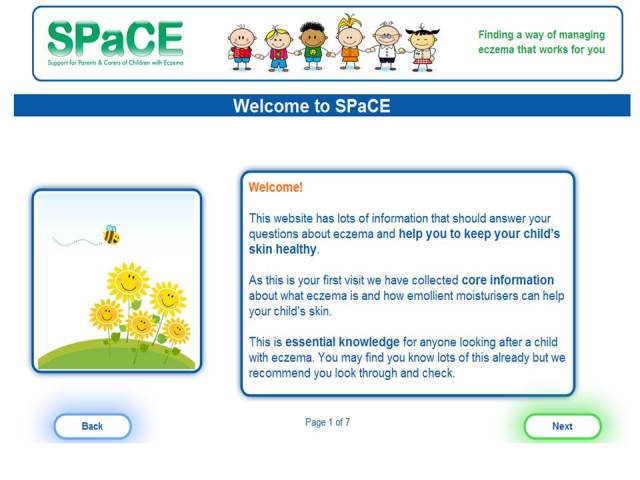
Screenshot of SPaCE website welcome page.

**Figure 2:**
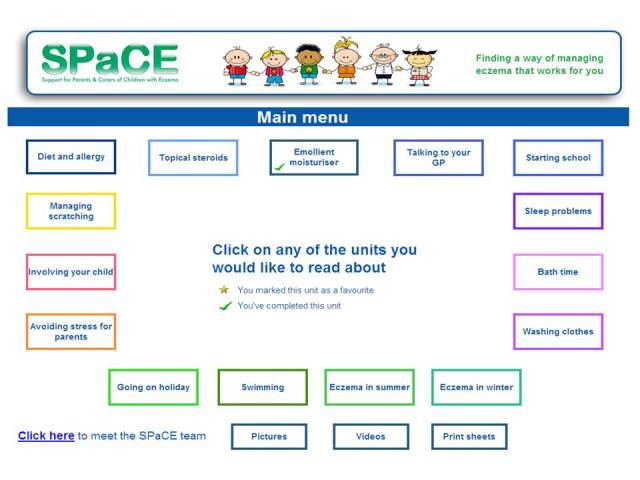
Screenshot of SPaCE menu of modules.

The intervention included 20 of 26 theory-based behavior change techniques listed in a taxonomy developed to standardize definitions [18] (Table 1). See Figures 1 and 2 for illustrative screenshots of the website. More screenshots are shown in Multimedia Appendix 1.

**Development of the Web-based intervention for childhood eczema.**

- We followed the PRECEDE-PROCEED model to devise the intervention [15] (ie, state the quality of life aim, create an inventory of potential target behaviors that might influence this, and factors that affect these behaviors). Three sets of factors are identified that can affect behavior: predisposing, reinforcing, and enabling.
- Our aim was to improve quality of life for families of children with eczema through better control of eczema.
- Our target behavior was regular emollient use because this is relevant to all children with eczema, is thought to be amenable to change, and there is clinical consensus about the benefits of regular use.
- Factors that influence regular emollient use include predisposing factors (knowledge, beliefs and attitudes, perceived control over performing behaviors), reinforcing factors (eg, delayed improvement with emollient use potential barrier to reinforcement), and enabling factors (eg, demonstrating techniques to make emollient application easier).
- We used LifeGuide software to build the intervention.
- We drew on findings from qualitative interviews [16,17], evidence-based patient information leaflets (Nottingham Support Group for Carers of Children with Eczema), and expertise from patient support groups and clinicians to inform intervention design.
- We knew that lack of time was a barrier for carers of young children with eczema so did not incorporate tailoring at the start of the intervention. Instead, all users were tunneled through 2 core modules before reaching a menu of modules to allow them to tailor the intervention to their own priorities.
- We sought feedback through think-aloud interviews with 17 parents of children with eczema who were asked to say all their thoughts and impressions of the website out loud while they were using a draft version. We obtained feedback via email from 3 additional parents and 4 health professionals.
- Changes were made iteratively on the basis of feedback.

**Overview of Web-based intervention for childhood eczema.**

- On their first visit to the website, users were tunneled through 2 core modules (“What is eczema?” and “Emollient moisturizers”) before reaching a menu of 14 modules to allow them to tailor the intervention to their own priorities. The menu of modules reflected the concerns of carers of children with eczema, including diet and allergy, topical corticosteroids, involving your child in treatment, bath time, sleep problems, and managing scratching. A full list can be viewed in the illustrative screenshots.
- On subsequent visits to the website, users would go straight to the menu of modules. After completing a module, they would be asked whether they wished to mark it as a favorite. A tick would appear by this module if they had completed it and a star would appear if they had marked it as a favorite, which could be seen on subsequent visits.
- Some of the modules included short videos (approximately 4 minutes each) illustrating techniques such as applying emollients or bathing a child. Links to these videos were contained in the relevant modules, but there were also links to all the videos in a button on the menu screen. Similarly, print sheets (eg, tick charts for children, action plan to take to GP consultation, summary of eczema management for relatives, school, or nursery) appeared in the relevant modules, but there were also links to all these in a button on the menu screen.

**Table 1:** Behavior change techniques [16] that are incorporated into the SPaCE Web-based intervention.

Technique	Definition	How is this addressed in SPaCE	Where technique is used in SPaCE
1. Provide information about behavior-health link	General information about behavioral risk (eg, susceptibility to poor health outcomes or mortality risk in relation to the behavior)	Information on role of emollients in controlling eczema and preventing flare-ups	Emollients module
2. Provide information on consequences	Information about the benefits and costs of action or inaction, focusing on what will happen if the person does or does not perform the behavior	Information on role of emollients in maintaining healthy skin and keeping child comfortable	Emollients module
3. Provide information about others’ approval	Information about what others think about the person’s behavior and whether others will approve or disapprove of any proposed behavior change	Telling other people’s stories	Emollients module, topical steroids module
4. Prompt intention formation	Encouraging the person to decide to act or set a general goal (eg, to make a behavioral resolution such as “I will take more exercise next week”)	Encourage participants to sign up to 2-week challenge where they can print out a chart and opt to receive daily email or SMS text reminders	Emollients module
5. Prompt barrier identification	Identify barriers to performing the behavior and plan ways of overcoming them	Acknowledge child resistance, telling other people’s stories	Involving your child module
6. Provide general encouragement	Praising or rewarding the person for effort or performance without this being contingent on specified behaviors or standards of performance	Telling other people’s stories of how regular emollient use has helped	Emollients module + throughout intervention
7. Set graded tasks	Set easy tasks and increase difficulty until target behavior is performed	—	
8. Provide instruction	Telling the person how to perform a behavior and/or preparatory behaviors	Videos demonstrating techniques for emollient application	Emollients module, bath time module
9. Model or demonstrate the behavior	An expert shows the person how to correctly perform a behavior (eg, in class or on video)	Videos demonstrating parent applying emollient to child	Emollients module, bath time module
10. Prompt specific goal setting	Involves detailed planning of what the person will do, including a definition of the behavior specifying frequency, intensity, or duration, and specification of at least 1 context (ie, where, when, how, or with whom)	Planning when/where/how to apply emollients during 2-week challenge	Emollients module
11. Prompt review of behavioral goals	Review and/or reconsideration of previously set goals or intentions	—	
12. Prompt self-monitoring of behavior	The person is asked to keep a record of specified behavior(s) (eg, in a diary)	Offering monitoring sheet for 2-week challenge	Emollients module
13. Provide feedback on performance	Providing data about recorded behavior or evaluating performance in relation to a set standard or others’ performance (ie, the person received feedback on their behavior)	—	
14. Provide contingent rewards	Praise, encouragement, or material rewards that are explicitly linked to the achievement of specified behaviors	Completion of each module receives congratulations and an extra tick or star; provision of star chart	Main menu; printable star charts for carer or child to use
15. Teach to use prompts or cues	Teach the person to identify environmental cues that can be used to remind them to perform a behavior, including times of day or elements of contexts	Advise use after bath, at nappy (diaper) changes, other set times	Emollients module, bath time module
16. Agree on behavioral contract	Agreement (eg, signing) of a contract specifying behavior to be performed so that there is a written record of the person’s resolution witnessed by another	—	
17. Prompt practice	Prompt the person to rehearse and repeat the behavior or preparatory behaviors	SMS text message at 6 pm every day during 2-week challenge	Emollients module
18. Use follow-up prompts	Contacting the person again after the main part of the intervention is complete	Participants received up to 4 reminder emails to log into SPaCE that contained prompts about how the website can help them	Emails
19. Provide opportunities for social comparison	Facilitate observation of nonexpert others’ performance (eg, in a group class or using video or case study)	Telling other people’s stories	
20. Plan social support or social change	Prompting consideration of how others could change their behavior to offer the person help or (instrumental) social support, including buddy systems and/or providing social support	Provision of printouts for other family members and/or nursery to help them understand need for regular emollient use	Emollients module summary printout
21. Prompt identification as a role model	Indicating how the person may be an example to others and influence their behavior or provide an opportunity for the person to set a good example	—	
22. Prompt self-talk	Encourage use of self-instruction and self-encouragement (aloud or silently) to support action	Identify negative thoughts and encourage alternative thoughts	Avoiding stress module
23. Relapse prevention	Following initial change, help identify situations likely to result in readopting risk behaviors or failure to maintain new behaviors and help the person plan to avoid or manage these situations	Discussing methods to avoid child resistance	Involving your child module
24. Stress management	May involve a variety of specific techniques (eg, progressive relaxation) that do not target the behavior but seek to reduce anxiety and stress	Stress management techniques	Avoiding stress module
25. Motivational interviewing	Prompting the person to provide self-motivating statements and evaluations of their own behavior to minimize resistance to change	—	
26. Time management	Helping the person make time for the behavior (eg, to fit it into a daily schedule)	Tips for fitting emollients into everyday life were included both in core information and in quotes from other peoples’ stories	Emollients module

### Study Population

Our intervention was aimed at carers of children with mild or moderate eczema; most of whom were managed in general practice in the United Kingdom. We recruited through a mail-out from 31 general practitioner (GP) practices in South West England, recruiting as many practices as possible from areas of social deprivation because this part of England is generally above average in terms of socioeconomic class. General practices posted invitations to parents/carers of children aged 5 years or younger with eczema. Carers were asked to post a reply to the research team if they wished to participate in the study.

### Inclusion/Exclusion Criteria

**Figure 3:**
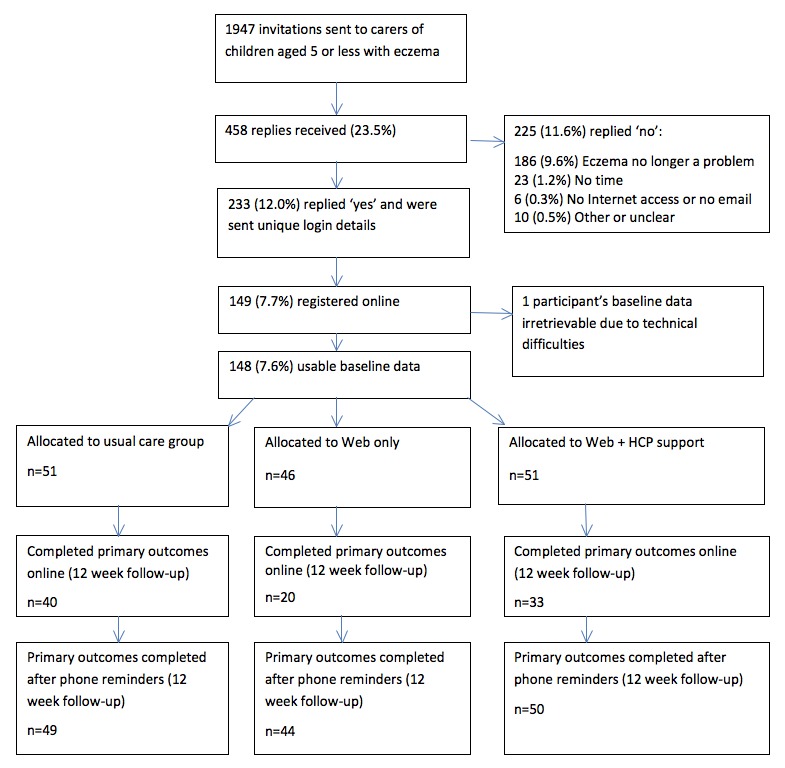
Flow of study participants through trial.

Inclusion criteria included parent/carer of a child aged 5 years or younger with a GP diagnosis of eczema who had obtained a prescription for this in the past year. Exclusion criteria applied by GPs included child aged older than 5 years, known severe mental distress, recent bereavement, opposition to involvement in research, carer unable to give informed consent, or with insufficient English to use website or complete outcome measures. If carers did not wish to take part, they were invited to indicate on the reply slip why this was (see Figure 3). If a family had more than one child meeting eligibility criteria, they were asked to choose one child when answering questionnaire items about their child’s eczema.

### Pilot Randomized Controlled Trial

If carers replied that they were interested in participating, they were sent a unique log-in to complete online consent, baseline questionnaires, and randomization. Online randomization was carried out using LifeGuide software allocating participants to 1 of 3 equal groups: (1) usual care plus Web-based intervention, (2) usual care plus Web-based intervention plus HCP support, or (3) usual care.

Emails were sent 12 weeks later requesting online questionnaire follow-up with 2 email reminders and subsequent phone follow-up for nonresponders requesting responses for core outcome measures.

Health care professional support was a single appointment aimed at engaging carers with the intervention, rather than teaching them about eczema. In 11 practices this was carried out by a practice nurse, but 1 practice assigned the role to a health care assistant and 1 to a GP. Only one of the practice nurses had specific dermatology training. Health care professionals were asked to spend up to 1 hour familiarizing themselves with the intervention. Participating HCPs were then asked to phone participants to arrange a 20-minute appointment at a convenient time in the first few weeks after randomization. The HCPs were informed that their role was not to deliver eczema care, but to encourage participants to use the website as a resource to help them manage their child’s eczema. They were asked to have the website open during the appointment and to go through the core modules with the participants if they had not already completed them, and to discuss the 2-week challenge if they had not already done this. If they had completed these, the HCPs were asked to allow the participant to choose other modules to go through together.

Usual care consisted of carers continuing to attend services in the usual way. For most participants this meant making an appointment with their GP when they felt it necessary. A minority were under regular secondary care review by a dermatologist or dermatology nurse.

### Outcome Measures

Our primary outcome was the Patient-Oriented Eczema Measure (POEM) [19] questionnaire completed online by carers at 12 weeks. This includes 7 questions about eczema symptoms over the previous week which are summed to give a score from 0 (no eczema) to 28 (worst possible eczema). POEM is a patient-reported outcome that can be used by proxy (carer report), is recommended by NICE, and demonstrates good validity, repeatability, and responsiveness to change [20]. Disease-specific quality of life was measured by the Dermatitis Family Impact (DFI) questionnaire, Infants Dermatitis Quality of Life (IDQoL) index, and Children’s Dermatology Life Quality Index (CDLQI). The DFI [21] is a widely used validated instrument that measures the impact of eczema on the family’s quality of life. The IDQoL [22] and CDLQI [2] are validated measures in children aged 4 years or younger and 5 years or older, respectively.

To explore the effectiveness of the intervention, the pilot study also included questionnaire items to measure adherence (self-reported emollient use) and attitudes that should predict adherence (instrumental and affective attitudes, perceived attitudes of others, and perceived control over adherence). We included the Problematic Experiences of Therapy Scale (PETS), used in previous studies to explore adherence to interventions [23,24]. The PETS asks participants to what extent they have been prevented from carrying out the intervention by socially acceptable reasons for noncompliance (eg, uncertainty about how to carry out treatment, lack of time) before asking about adherence to the intervention and to treatment (emollient use). We also asked participants to complete the Patient Enablement Instrument, which assesses whether they feel they can understand and manage treatment better [25].

### Quantitative Analysis

A change in POEM score of 3 or more is thought to be clinically significant in a secondary care setting [26]. However, baseline POEM scores in primary care are lower because many children experience mild eczema; therefore, we determined a score change of 2 would likely to be a clinically significant difference. We wished to be able to detect a small difference because the intervention is relatively inexpensive and even small effect sizes are likely to be cost-effective for such a common condition. Differences between groups were examined using chi-square tests. Analyses were carried out on an intention-to-treat basis. Analyses were carried out using SPSS version 21 (IBM Corp, Armonk, NY, USA).

### Qualitative Interviews With Health Care Professionals

Qualitative interviews were carried out with 13 of 14 HCPs who provided support sessions, but one could not be contacted after repeated attempts. Interviews were carried out by phone by a researcher who was not otherwise involved in the study. The interview guide included general questions about their experiences of participating in the study and detailed questions about the conduct and content of appointments they had had with carers, including what parts of the website they had looked at, problems encountered at any stage, and their impressions of usefulness of the appointment for carers. Interviews were recorded, transcribed, and analyzed thematically.

### Qualitative Interviews With Participants

Of the 143 parents/carers who took part in the pilot study, 82 agreed to be contacted for a feedback interview. We approached 45 of these after they had completed their follow-up questionnaire, purposively sampling those who had been in the website only or website plus HCP groups, including those who had not attended appointments. We also purposively sampled participants from lower socioeconomic class areas. Of the 45 we approached, 14 were uncontactable after 6 attempts, 4 declined to be interviewed, 1 gave email feedback, and 26 were interviewed. We offered a choice of telephone or face-to-face interview; 10 interviews were carried out by phone, 15 were carried out in participants’ homes, and 1 at a participant’s workplace. Interviews lasted from 20 to 60 minutes and were audio-recorded, transcribed, and analyzed thematically.

### Qualitative Analysis

Thematic analysis was used to analyze the data [27]. Transcripts were read and reread to allow familiarization with the data. Initial coding was applied by MS then developed iteratively until all important information within the dataset was coded. Coding schedules and themes were further refined through discussion (MS, IM, and LY for HCP interviews; MS, HS, and LY for participant interviews). Coding was applied using NVivo 10 software to facilitate data handling. Pseudonyms have been assigned in reporting the findings.

### Ethics Approval

Ethical approval for this study was given by the Berkshire Research Ethics Committee (MREC: 10/H0505/56) as a registered randomized controlled trial (ISRCTN 98560867).

## Results

### Recruitment and Participant Characteristics

A total of 1947 invitation letters were sent from 31 GP practices to parents or carers of children with eczema. We received replies from 458 households (23.52%), with 233 of 1947 (11.97%) replying that they wished to take part in the study. The main reason for not wishing to take part was that their child’s eczema was no longer a problem (186/1947, 9.55%). In all, 149 of 1947 (7.65%) people registered online and were randomized into the trial, but 1 participant did not have usable data because of technical problems and was not included in the analysis. At follow-up, 88 of 143 (61.5%) completed the questionnaire online, 50 (35.0%) completed key questions by phone (POEM, DFI, and questions about adherence to emollient therapy), and 5 (3.5%) were uncontactable. Follow-up questions not asked by phone received response rates below 60%; therefore, they will not be presented here. Figure 3 shows the flow of participants through the study and Table 2 shows participant characteristics.

**Table 2:** Participant characteristics.

Participant characteristic		Website (n=46)	Website plus HCP (n=51)	Usual care (n=51)	Total (N=148)
**Gender of carer, n (%)**					
	Female	44 (96)	50 (98)	50 (98)	144 (97)
	Male	2 (4)	1 (2)	1 (2)	4 (3)
**Age of carer (years), n (%)**					
	≤25	1 (2)	3 (6)	3 (6)	7 (5)
	26-30	7 (15)	7 (14)	6 (12)	20 (14)
	31-35	12 (26)	17 (33)	18 (35)	47 (32)
	36-40	15 (33)	13 (25)	19 (37)	47 (32)
	41-45	10 (22)	10 (20)	2 (4)	22 (15)
	≥46	1 (2)	1 (2)	3 (6)	5 (3)
**Age of child (years), n (%)**					
	0	1 (2)	3 (6)	9 (18)	13 (9)
	1	9 (20)	11 (22)	11 (22)	31 (21)
	2	9 (20)	6 (12)	8 (16)	23 (16)
	3	11 (24)	14 (27)	13 (25)	38 (26)
	4	8 (17)	5 (10)	8 (16)	21 (14)
	5	8 (17)	12 (24)	2 (4)	22 (15)
**Age carer left education (years), n (%)**					
	15-16	7 (15)	8 (16)	6 (12)	21 (14)
	17-18	11 (24)	9 (18)	8 (16)	28 (19)
	19-21	12 (26)	19 (37)	18 (35)	49 (33)
	≥22	16 (35)	15 (29)	19 (37)	50 (34)

### Intervention Use

Total time spent on the website ranged from 8 to 253 minutes with a median of 40 minutes (IQR 23-59). Of the 93 participants in the website groups, 75 (81%) completed the core modules. This was completed with relatively few visits, with only 45 of 93 (48%) participants in the website groups visiting 3 times or more. Key components of the intervention were thought to be watching videos and signing up to the 2-week challenge (see Table 1), but only 35% of website users watched 1 or more videos and only 39% signed up for short message service (SMS) text message alerts for the 2-week challenge. There were no consistent differences in website use between the website only and the website plus HCP groups (Table 3).

Uptake of HCP support for the intervention was lower than expected. Only 23 of 50 (46%) participants who were allocated to the website plus HCP support group actually attended their appointments (12 declined the appointment, 9 participants could not be contacted to arrange an appointment, 6 made an appointment but did not attend).

**Table 3:** Intervention use.

Measures of website use	Website only (n=44)	Website plus HCP (n=49)	Website groups combined (n=93)
Total time spent on website (minutes), median (IQR)	34 (20-50)	45 (26-70)	40 (23-59)
Core modules completed, n (%)	38 (86)	37 (76)	75 (81)
3 or more visits to website, n (%)	16 (36)	29 (59)	45 (48)
Watched 1 or more videos, n (%)	16 (36)	17 (35)	33 (35)
2-week challenge SMS text alerts, n (%)	18 (41)	18 (37)	36 (39)

### Primary Outcome

Baseline POEM scores were relatively low (mean 9.0, SD 6.6), with POEM scores of 0-2=clear/almost clear, 3-7=mild, 8-16=moderate, 17-24=severe, and 25-28=very severe [28]. All groups showed improvement by 12 weeks (mean 7.8, SD 6.6) (see Table 4). This improvement was greater in the website groups: there was a decrease of 2 or greater in follow-up compared with baseline POEM score in 23 of 42 (55%) participants in the website only group, 16 of 49 (33%) in the usual care group, and 18 of 47 (38%) in the website plus HCP group (*P*=.09). The mean change in POEM score between baseline and follow-up was 1.56 in the combined website groups and 0.41 in the usual care group (ie, a difference between groups of 1.15, 95% CI –0.81 to 2.3).

To assess whether the intervention was more effective in those with higher baseline eczema severity, we restricted the analysis to the 96 participants with a baseline POEM score of 5 or more, but found a similar relationship: POEM score decreased by 2 or more in 21 of 30 (70%) participants in the website group, 17 of 35 (49%) in the website plus HCP group, and 13 of 31 (42%) in the usual care group (*P*=.07).

We also wished to look at whether duration of eczema diagnosis influenced effectiveness of the intervention, but only 14 of 130 (10.8%) participants who answered this question reported that their child had had eczema for 6 months or less. This subgroup was too small to allow reliable comparisons to be made.

**Table 4:** Patient-Oriented Eczema Measure (POEM) and Dermatitis Family Impact (DFI) scores at baseline and at 3-month follow-up.

Outcome measure		Usual care n=49	Website n=44	Website plus HCP n=50	Total n=143
**POEM** ^a^ **score, mean (SD)**					
	At baseline	7.47 (6.2)	10.3 (7.0)	9.4 (6.2)	9.01 (6.6)
	At follow-up	7.1 (6.6)	7.6 (6.1)	8.7 (7.0)	7.8 (6.6)
**DFI** ^b^ **score, mean (SD)**					
	At baseline	5.2 (5.9)	5.3 (5.3)	6.4 (5.6)	5.5 (5.6)
	At follow-up	4.4 (5.5)	4.0 (4.2)	5.9 (5.3)	5.0 (5.1)

^a^POEM includes 7 items scoring 0, 1, 2, 3, and 4 to give a total score out of 28, where 28 is most severe.

^b^DFI includes 10 items scoring 0, 1, 2, and 3 to give a total score out of 30, where 30 is most severe.

### Secondary Outcomes

The DFI showed even lower baseline scores than POEM. The mean baseline score in this study was 5.7 (SD 5.6), and mean follow-up DFI was 4.8 (SD 5.1). This score is too low to demonstrate differences between groups because of floor effects.

### Health Care Provider Feedback

Qualitative interviews were carried out with 13 HCPs, 11 practice nurses, 1 GP, and 1 health care assistant. All were women except for the GP. Our findings centered around the following themes: HCP apprehensions about taking part in the study, HCPs and the self-care support role, and HCP perceptions of the usefulness (or not) of the appointments. HCPs said they were pleased to be involved in a study that gave them the opportunity to provide a self-care support role. However, they did report feeling apprehensive initially about supporting self-care for a condition in which they did not view themselves as expert. Training was viewed as very minimal and some concerns remained after training. After seeing participants, HCPs thought consultations had gone well and felt supported by the website, saying that they liked having it open during consultations and felt this worked well. HCPs observed that some parents seemed to find the HCP session more useful than others and felt their support to be unnecessary in some cases:

I think my only concern really was that they were going to expect me to know a tremendous amount about eczema, and that I wouldn’t sort of meet their expectations...but that didn’t really happen to be honest, because obviously all of the answers were on the website so they were in front of me as well as them.

Geena

I thought it was good they could point at, for instance use of steroids, and...discuss it in further detail. So they were able to pick an aspect that was from a third party, which is the website, and then bounce ideas or bounce queries off of me. And I think looking to the future, if people have that knowledge already, that might be something that they would be able to use more effectively in a consultation.

William

The one lady definitely liked the support, the other lady was sort of a bit like, well I’ve done it, why do I need to come in and talk to you about it?

Qadira

### Participant Feedback

Two men and 24 women, ages ranging from 24 to 44 years, were interviewed. Five were full-time carers and the remainder were working full-time or part-time in a wide range of occupations. Most parents interviewed were managing mild eczema. They had all been managing their child’s eczema for at least 6 months, but most had been managing it for some years.

Most people found the website helpful, easy to use, and easy to understand. The benefits they ascribed to website use are shown in Table 5.

Five of the 26 interviewees said they did not find the website helpful, either because it would have been helpful in the past but that they were confident in managing their child’s eczema now or because their child’s eczema was so mild that it did not feel relevant. However, there were others managing mild or longstanding eczema who still found the website helpful. So, although confidence in self-management appeared to be related to how useful they found SPaCE, duration of diagnosis and severity were not necessarily important.

**Table 5:** Qualitative findings from participant interviews about experiences of using SPaCE website.

Theme	Summary	Illustrative quote
Confidence in eczema management	Approximately one-third of participants said using SPaCE had made them more confident in managing eczema, more able to control it, or more confident that they would be able to manage future flare-ups	*The website was very useful and then from that, it’s kind of changed some of our approaches to-to how we treat the eczema of our youngest daughter. It’s made us more confident actually, I’d say.* [David]
Getting into the habit of using emollients	Approximately half of interviewees said they had increased emollient use since using the website, mainly related to the 2-week challenge. 3 interviewees mentioned the (optional) daily text reminders and had really liked this. Benefits ascribed to the 2-week challenge included demonstrating that emollient helped; demonstrating that emollient did not help, leading family to try a new emollient; establishing habit of regular emollient use; helping child to accept regular emollient use	*We printed out the 2-week trial tick sheet. He thought it was good, cos it involved him [because] he got to have a tick if he put his cream on which I think helped him with it. Because sometimes he doesn’t like it because the creams can be quite thick, so that helped, having a reward chart.* [Jo]
Increased awareness of treatments available or confidence in treatments available	Some carers said that the website had made them aware that there were different treatments available for eczema, for instance number of different types of emollient and that it may be worth trying different ones. Some said that the website had allayed their fears about using topical corticosteroids on their child’s skin	*We’ve only ever had Diprobase and hydrocortisone cream, but apparently there are lots more emollients that you can use, which I didn’t realize. So I was wondering-I’ll keep going with the Diprobase for a bit but actually if we are still not getting any improvement, perhaps go and speak to the doctor and get another one.* [Annie]
Views about consulting the GP	Some carers said that using the website had made them feel more confident to re-consult if the eczema was not improving. Others said that they felt the website would help them to consult less as they could use the website for reference if they had questions about eczema care in the future	*I thought the creams weren’t working, I just sort of thought, “Oh, creams aren’t any good for this sort of thing.” But it made me realize that I could actually just keep going back to the doctor and not feeling like I was just being an overprotective mother and sort of like complaining about things. So it gave me confidence to actually sort of approach the doctor with my concerns about my son.* [Hayley]
Videos	Videos led to increased awareness of techniques for applying emollients, particularly using larger volumes	*What I actually found useful was the videos because, to be honest, I just used to put on the cream and I obviously wasn’t putting on enough on and just going through those little videos, it showed me I needed to step up on the amount I was putting on. * [Bee]
“It’s nice to know you’re not alone”	Carers valued increased awareness that they were not alone in finding eczema management challenging and that their child was not alone in resisting the application of creams	*I just think it is really helpful: again, it just reminds you that there are other people going through the same sorts of things. Because it can, you know, it’s one of these things where it’s not the worst thing in the world, some children have terrible illnesses, but in the middle of the night when your child is absolutely crying in agony because her skin hurts so much and they’re bleeding through the pajamas, you just think, “Oh, this is just really difficult.” But knowing that there are going to be other people in a similar situation...it’s kind of reassuring.* [Emily]

### Feelings About Being Offered Health Care Provider Support

In the website plus HCP group, 15 of 26 were interviewed, of whom 10 had attended appointments and 1 had discussed her daughter’s eczema by phone with the practice nurse. Of the remaining 4, 1 forgot to attend, 1 did not think she had been contacted by the practice, 1 said that the times offered by the nurse were unsuitable for her, and 1 declined the appointment. Sophie was offered a HCP appointment but did not attend:

I might have done if he was younger and I was still in the state of, “I don’t know what I’m doing and am I doing it right,” but I think, because I feel like I know how it’s going that I didn’t really need any more help really. I think the website being there put me at ease, where if I’d have had no website and the nurse had rung, I would probably have gone to see her, if you see what I mean, because it was nice to have reassurance.

Sophie

Kim had a nurse appointment that she forgot to attend:

I wanted to go and see her and tell her that I want to help the study, but like I didn’t, it sounds really conceited, but I didn’t think I would be told anything I didn’t know. Maybe I would have done, like I did in the modules, but I wasn’t really thinking it was going to be any great difference in what I already know.

Kim

The 11 interviewees in the website only group were mainly happy with their group allocation, saying that the website was enough or fit with everyday life better than attending a nurse appointment. Only 2 of 11 interviewees said that they would have liked to have been offered an appointment: one offered no reason and the other said it might have made her more enthusiastic about using the website more.

### Experiences of Participants Who Attended Health Care Provider Support Session

Parents’ views regarding the value of the HCP support varied widely. Of the 10 who attended HCP appointments, 4 said they found the appointment useful (although one of these discussed food allergies rather than eczema), 3 said they did not find it useful, and the remainder were noncommittal about whether they found it useful or not. Reasons parents gave for not valuing the HCP appointment included not really feeling they needed help with eczema management (eg, parents felt confident in managing it) or that they felt confident in obtaining information through the Internet:

We did go and see a nurse during the trial but she didn’t say anything different to what I already knew anyway, so when we made the appointment and went up to see her I must admit, I did think, “Why am I here?”

Charlotte

Among the few carers who found the HCP appointment useful, they reported that this had encouraged them to revisit the website or directed them to part of website they had not looked at before (eg, videos) or helped them to feel more confident about consulting in the future (although other participants ascribed this to the website rather than the nurse appointment). One participant had enjoyed her appointment, but had chosen to discuss health problems other than eczema and another valued her appointment because she was provided with emollient samples (although this was not part of the intervention):

I met with the nurse practitioner at my GP surgery and she went through a couple of things on the website with me and we discussed some things together. So actually it brought me closer to the medical team within my own GP surgery as well, so the website was a bit of a connection...and, you know, it’s kind of-very much a long-term situation with eczema but at least I feel like I know who I can speak to if I need to get advice.

Emily

Nicole is unusual in our sample in that she did not feel comfortable with the Internet and, therefore, really valued the nurse input:

To be honest I’m not a massive kind of computer person, I don’t work with computers...So it’s probably more to do with that I’m not really used to using them, I don’t really use computers in my daily life. So I think just going and speaking to the nurse was more helpful to me personally.

Nicole

## Discussion

### Principal Results

This pilot RCT showed a greater improvement in carer-reported eczema scores in both the website only and website plus HCP groups compared with a usual care group. Although the study was not powered, potentially important changes in outcomes were observed in the hypothesized direction and, although not significant at the 5% level, they were significant at the 10% level. Completion of core modules was good in both the website only and the website plus HCP groups, although uptake of some of the intervention components was low.

This study showed the feasibility of HCPs using a website to support self-care for eczema within the consultation and that initial concerns about providing support for a condition they were not expert in dissipated after they had tried it. However, among this group of carers it seems that many did not particularly value the HCP support, as evidenced by the low take-up of support session appointments and the qualitative findings.

### Limitations

Our initial mail-out received a low response rate; therefore, those using the intervention could be unrepresentative of families of children with eczema, possibly with higher health literacy or higher motivation than other families. Our intervention, if shown to be beneficial, is designed for dissemination to families of children newly diagnosed with eczema in primary care. Ideally, we would have carried out opportunistic recruitment of newly diagnosed families, but this would not be feasible in primary care so we recruited by mail-out instead, with the consequence that the intervention was less relevant to some families, probably contributing to the low response rate.

The relatively low use of some components of the intervention (videos and 2-week challenge) suggests that we need to refine the intervention before further evaluation. Although the intervention was developed carefully and adapted on the basis of initial feedback, complex interventions and trials to examine their efficacy frequently need further iterative development [29].

We are unable to comment on adherence to the target behavior (emollient use) for this intervention because these findings are extremely difficult to interpret. If eczema is in remission, then twice-daily emollient is adequate, but if there is a flare up, then more frequent application is required. Therefore, increased use of emollient can either be related to better adherence or worse eczema, and decreased use of emollient could mean worse adherence or better eczema. For instance, if a child was no longer bathing in soapy water as a result of the intervention, this could lead to an improvement in eczema and effective self-care could mean that emollient use stays the same or even decreases.

### Comparison With Prior Work

Other studies have found that human support is important in engaging users with online interventions [14,30], but we did not find that in this study. Unsupported use of Web-based interventions requires a fairly high degree of critical health literacy and motivation (ie, due to high symptom distress) [31]. Qualitative interviewees in our study were predominantly comfortable obtaining information online and often referred to the mildness or severity of eczema as being important in how motivated they were to engage with the website. It may be that there are particular features of the population under study that means they are less likely to value health professional support, such as that they were adults aged 20 to 40 years (so relatively computer literate), that eczema is a condition in which a high degree of self-care is expected [7], and many were looking after relatively mild eczema. It is also possible that participants would have welcomed the support more if they had initiated contact with the HCP themselves, rather than the reverse.

### Conclusions

We conclude that a full-scale RCT of this Web-based intervention is feasible. The findings presented here suggest that HCP support for this study population might not be necessary; therefore, this will not be included in the RCT. These findings also support keeping the invitation to participate quite broad because some parents caring for mild or longstanding eczema still valued having access to the website (even though it was targeted at the newly diagnosed). The website will be further refined before the next stage of evaluation.

## Multimedia Appendix 1

Illustrative screenshots.

## Abbreviations

CDLQI: Children’s Dermatology Life Quality Index
DFI: Dermatitis Family Impact
GP: general practitioner
HCP: health care professional
IDQoL: Infants Dermatitis Quality of Life
PETS: Problematic Experiences of Therapy Scale
POEM: Patient-Oriented Eczema Measure
RCT: randomized controlled trial
SMS: short message service
SPaCE: Supporting Parents and Carers of children with Eczema
