# Supplementary figures and images for: Supporting Self-Care for Families of Children With Eczema With a Web-Based Intervention Plus Health Care Professional Support: Pilot Randomized Controlled Trial
Source: J Med Internet Res. 2014 Mar 4;16(3):e70. doi: 10.2196/jmir.3035 (PMC3961807; doi:10.2196/jmir.3035)

## Slide 1
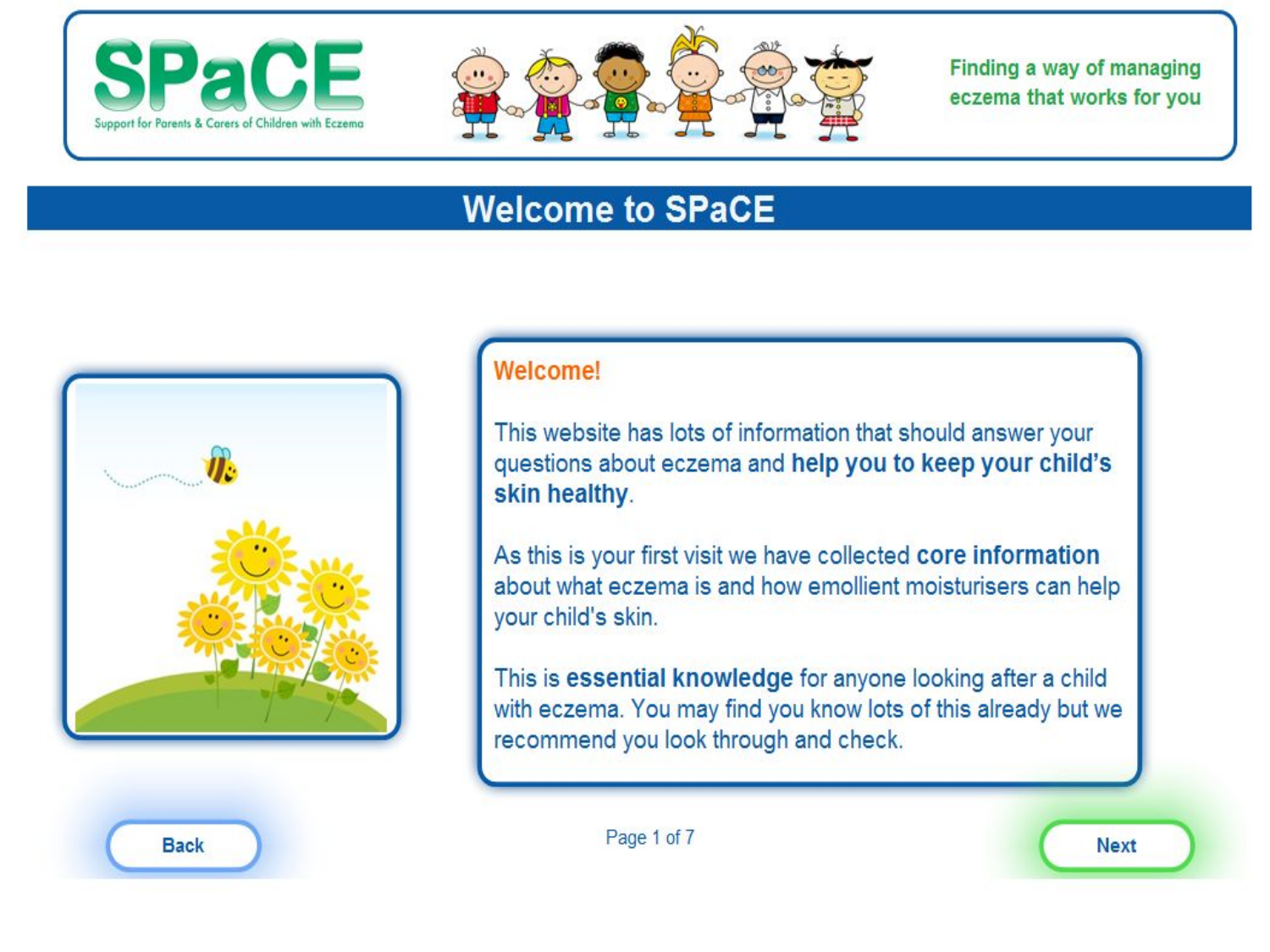

## Slide 2
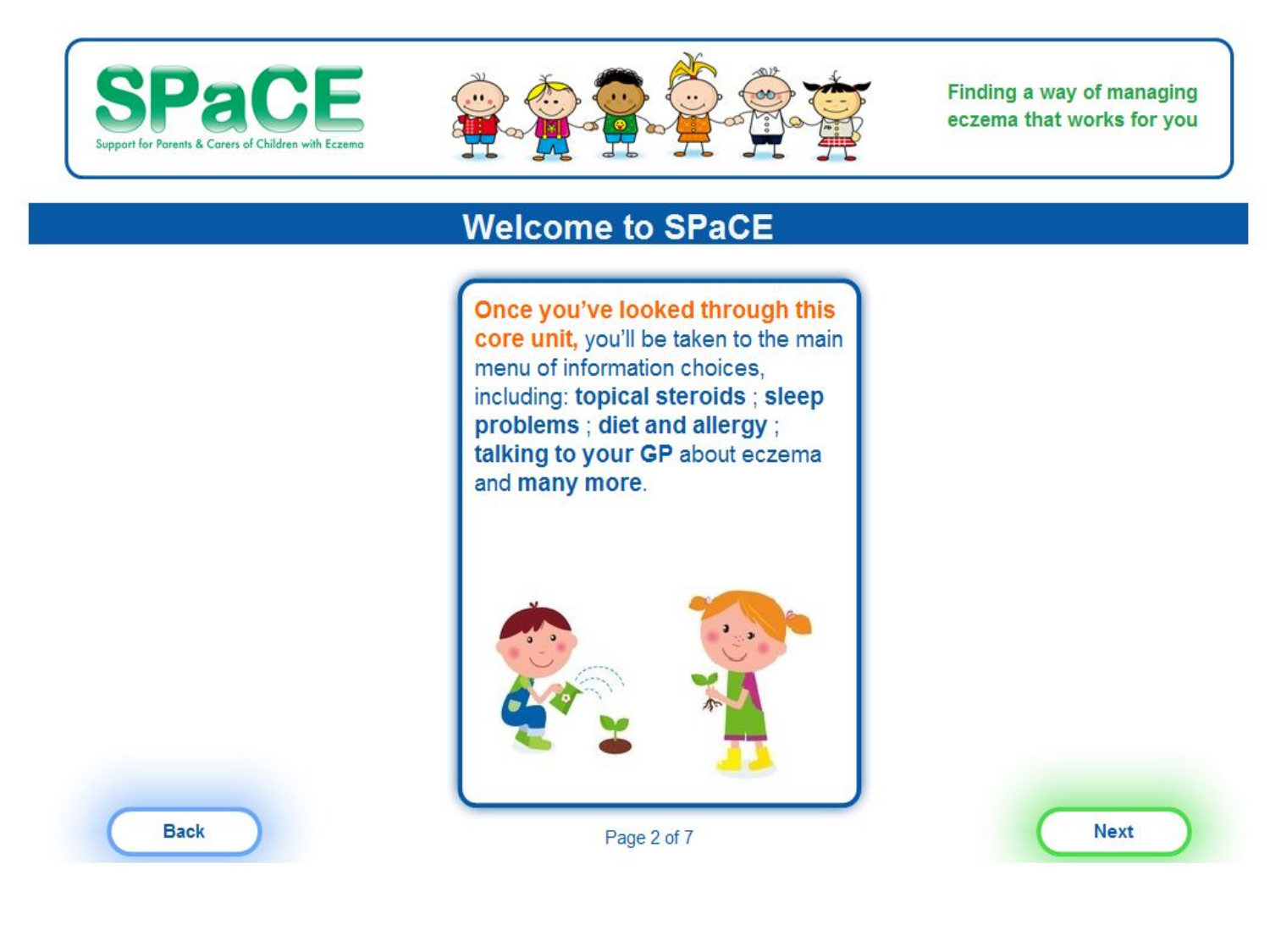

## Slide 3
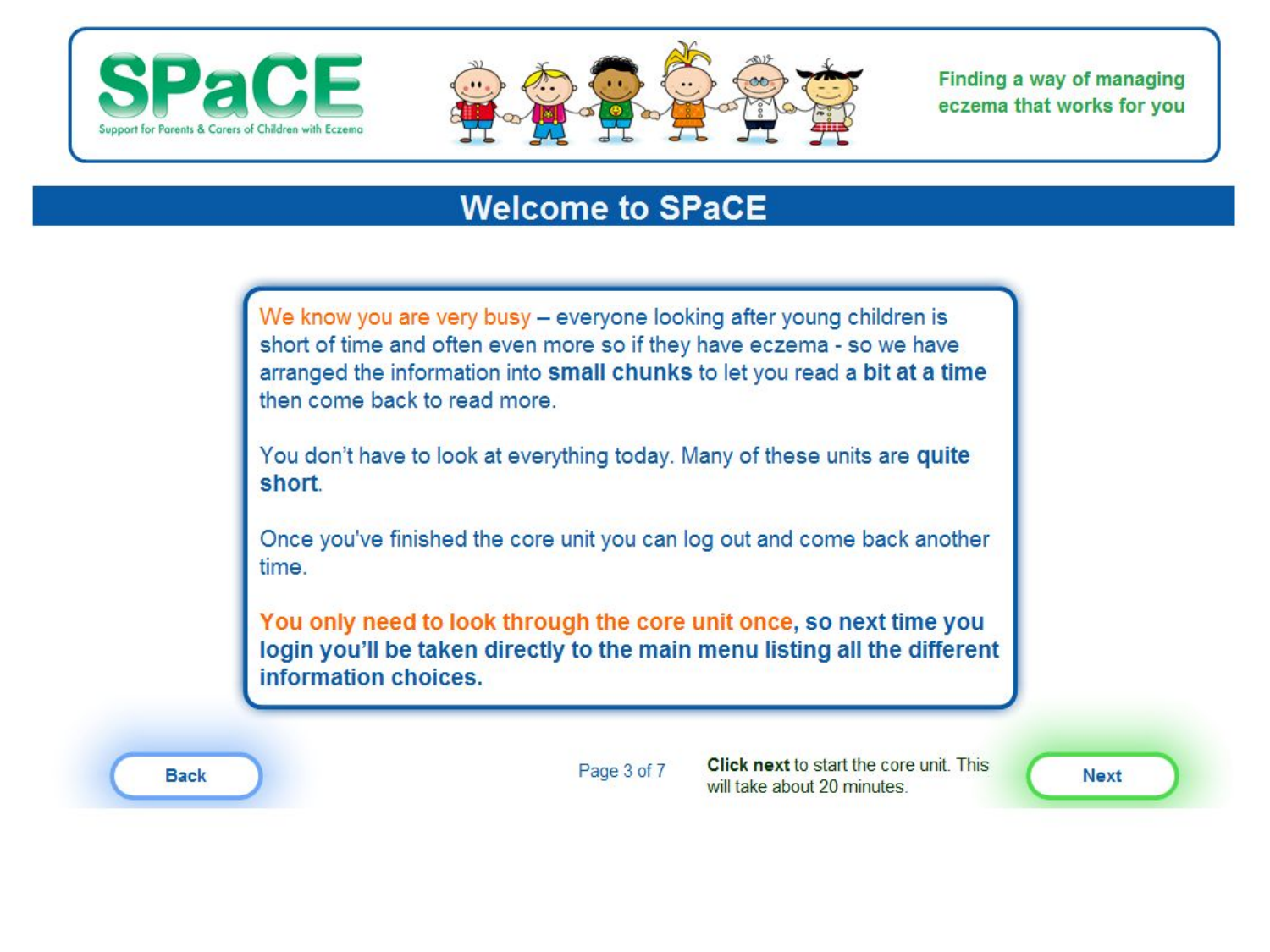

## Slide 4
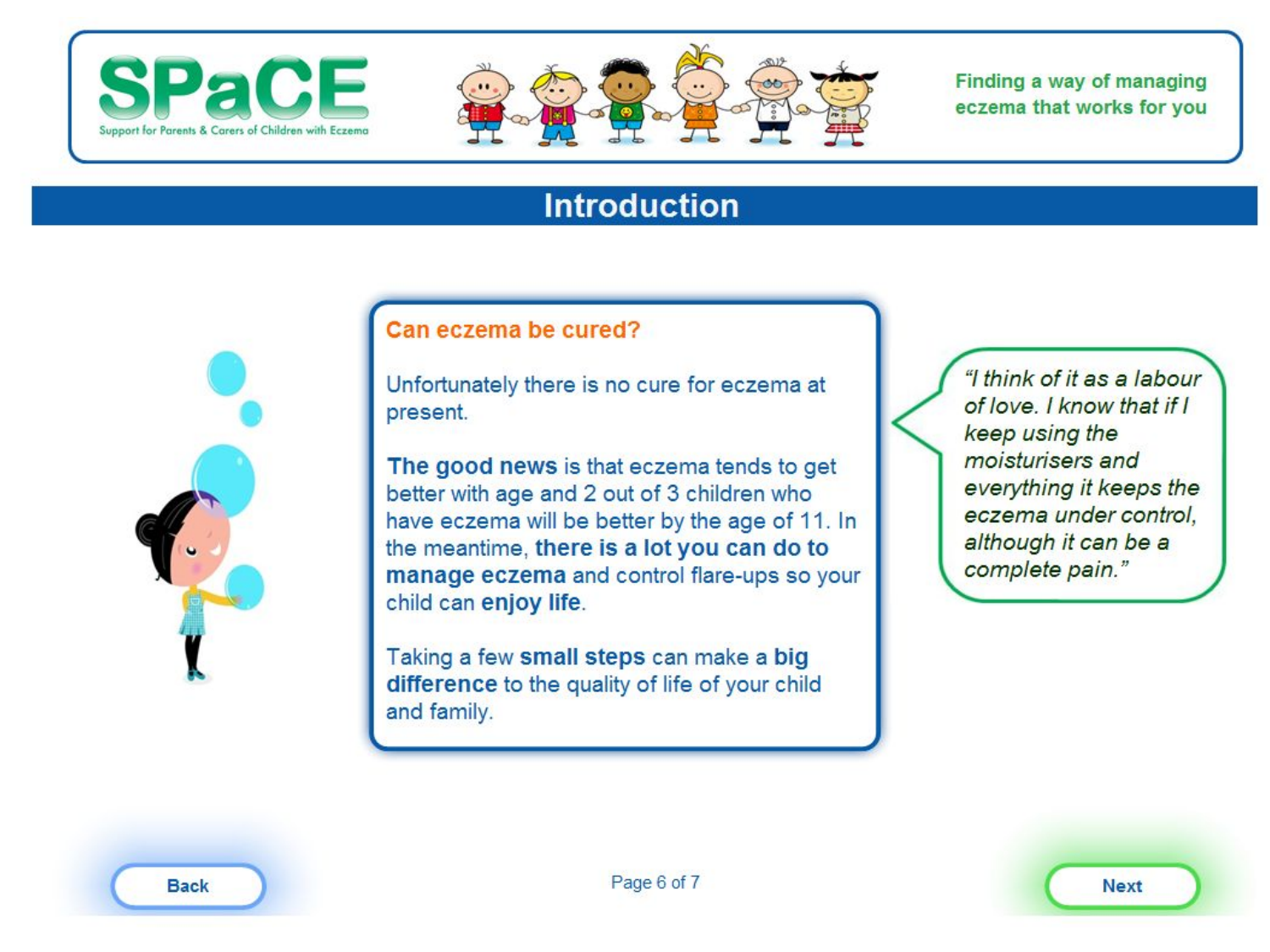

## Slide 5
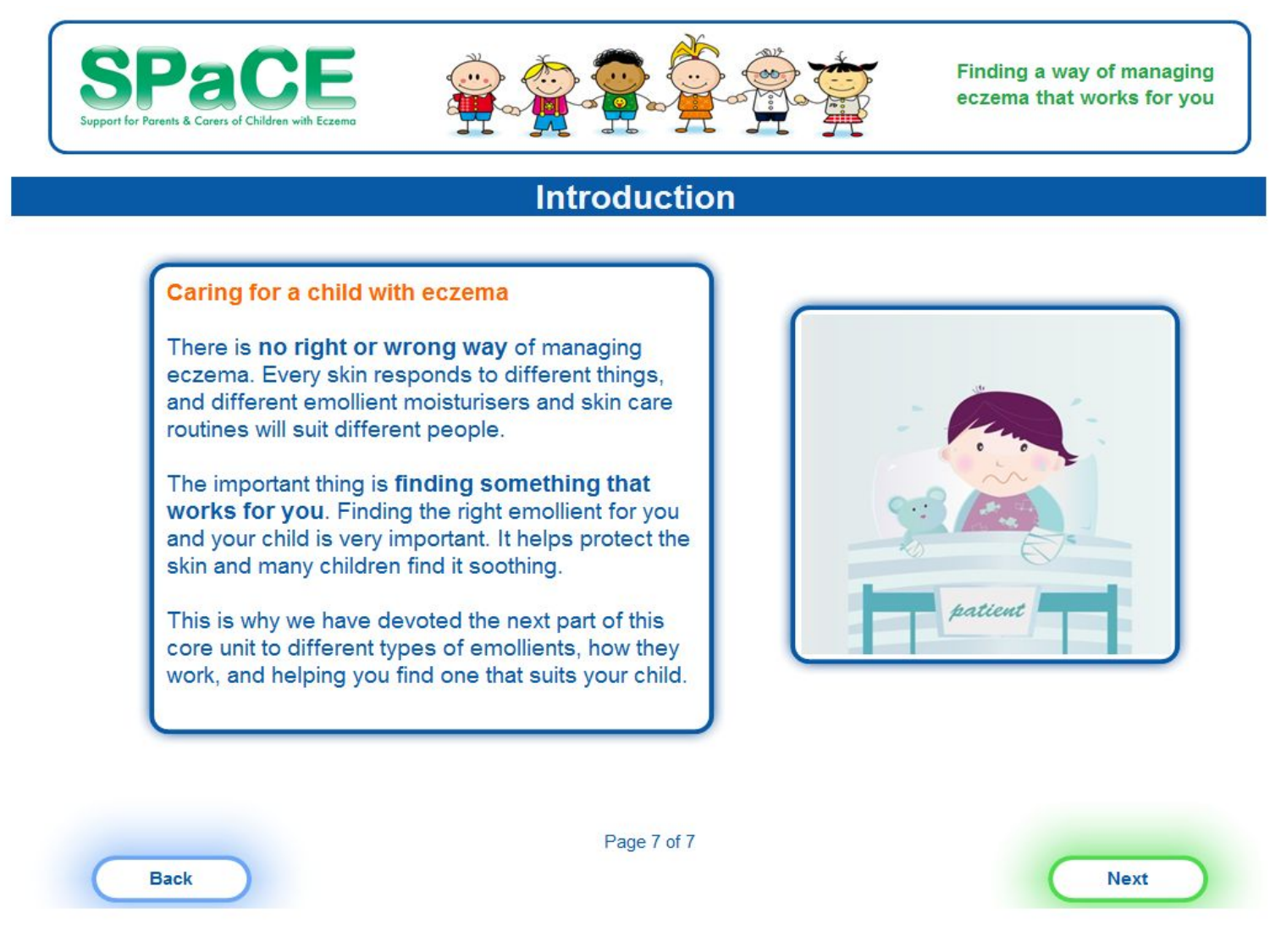

## Slide 6
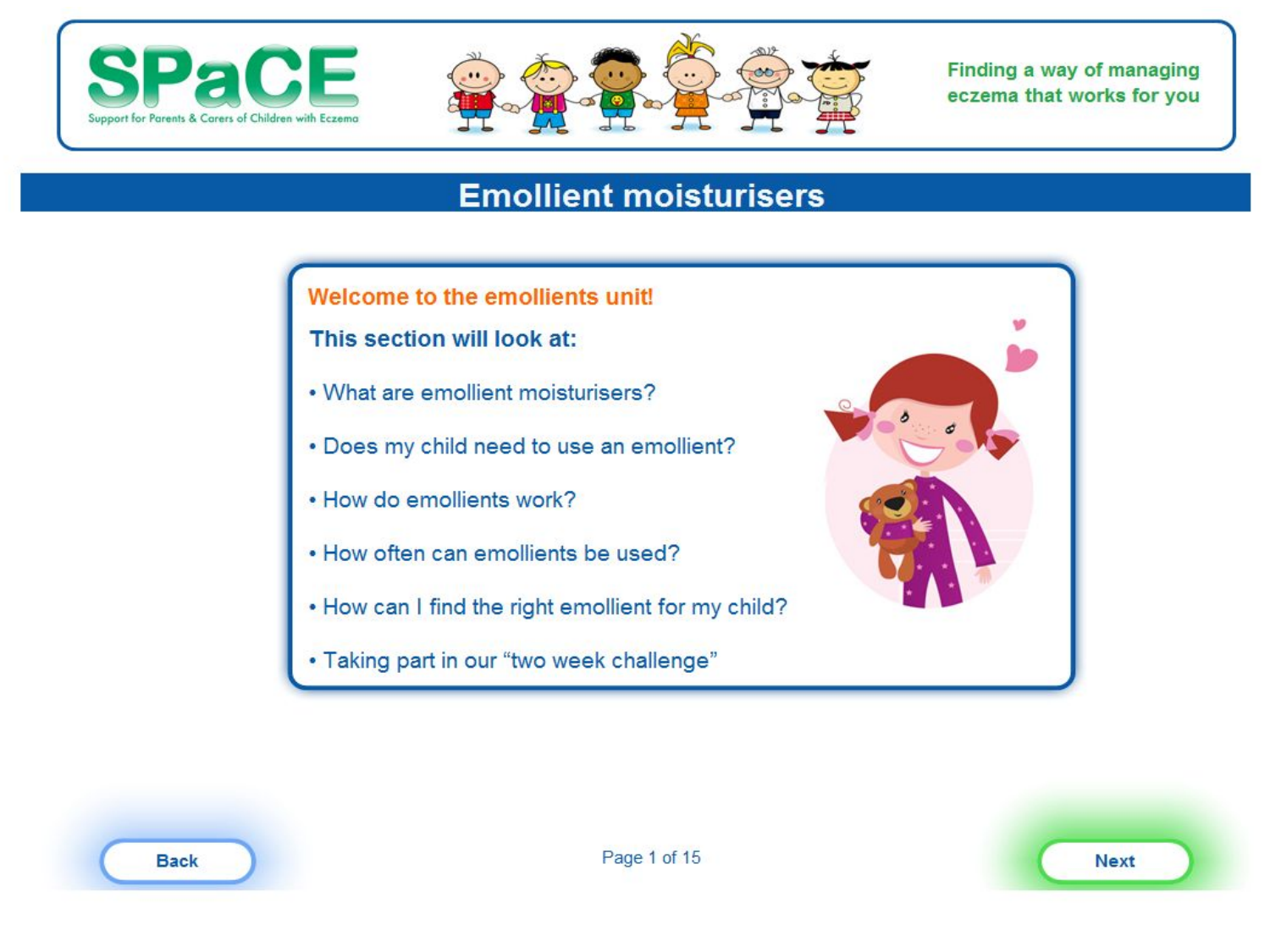

## Slide 7
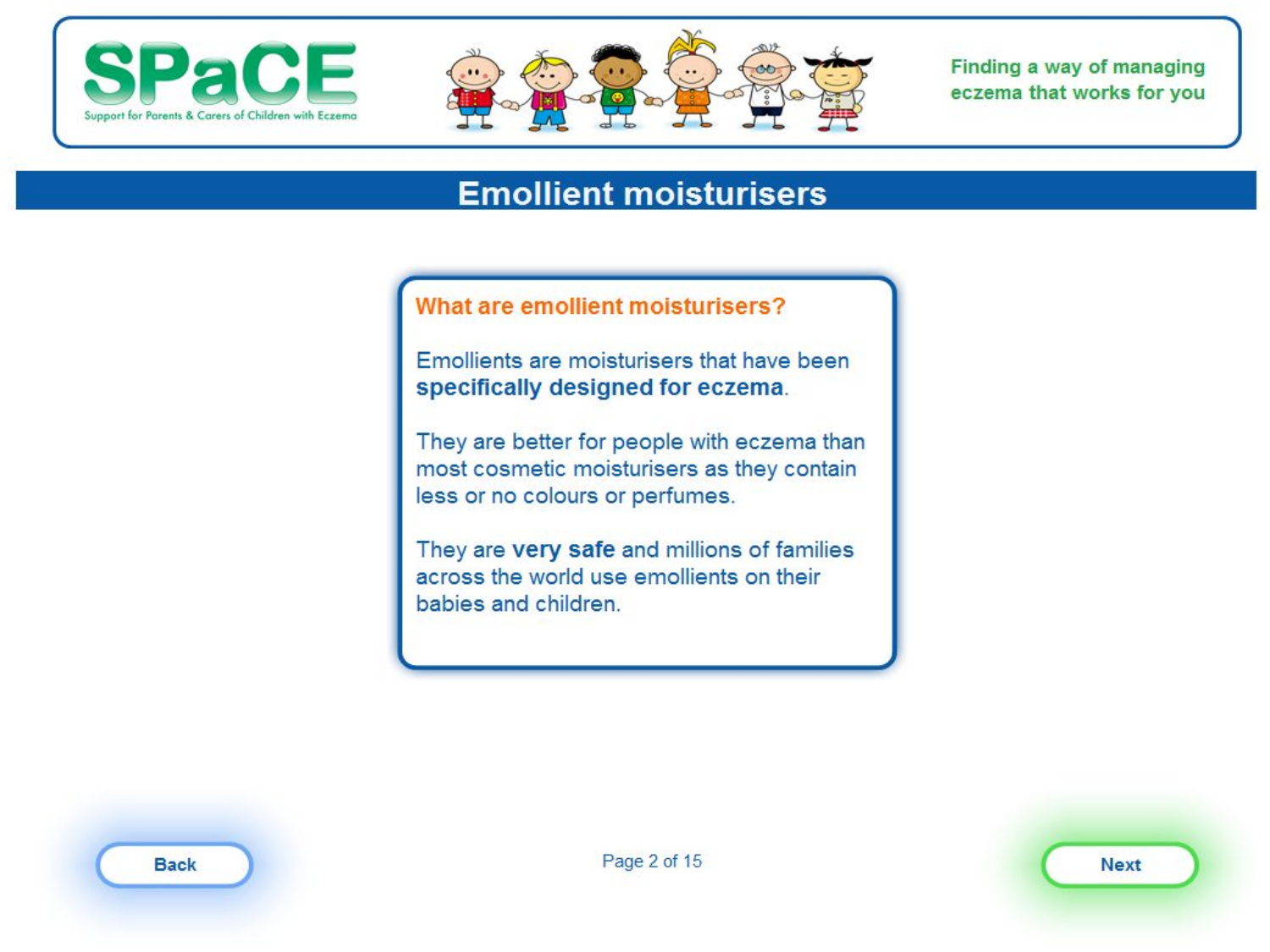

## Slide 8
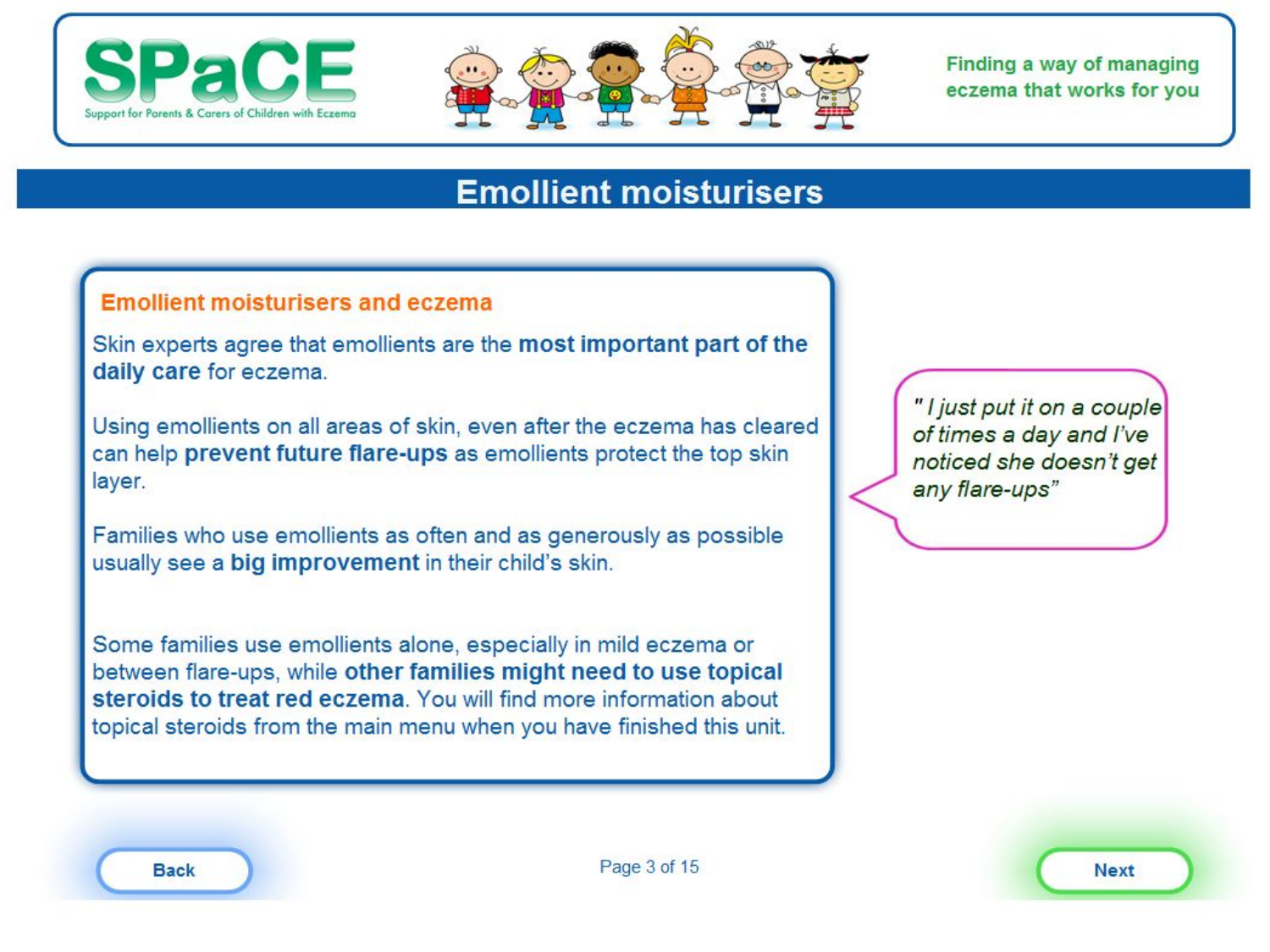

## Slide 9
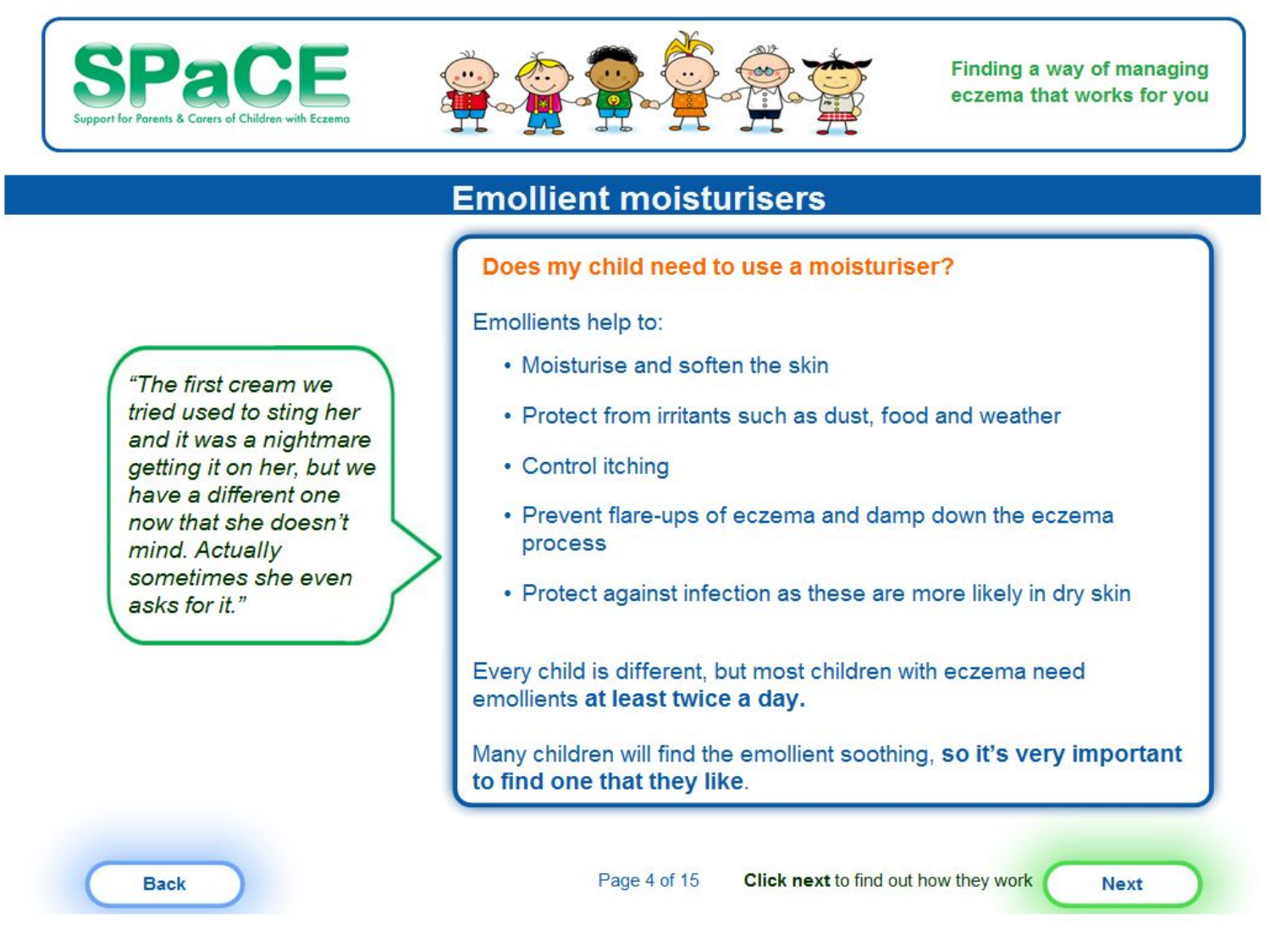

## Slide 10
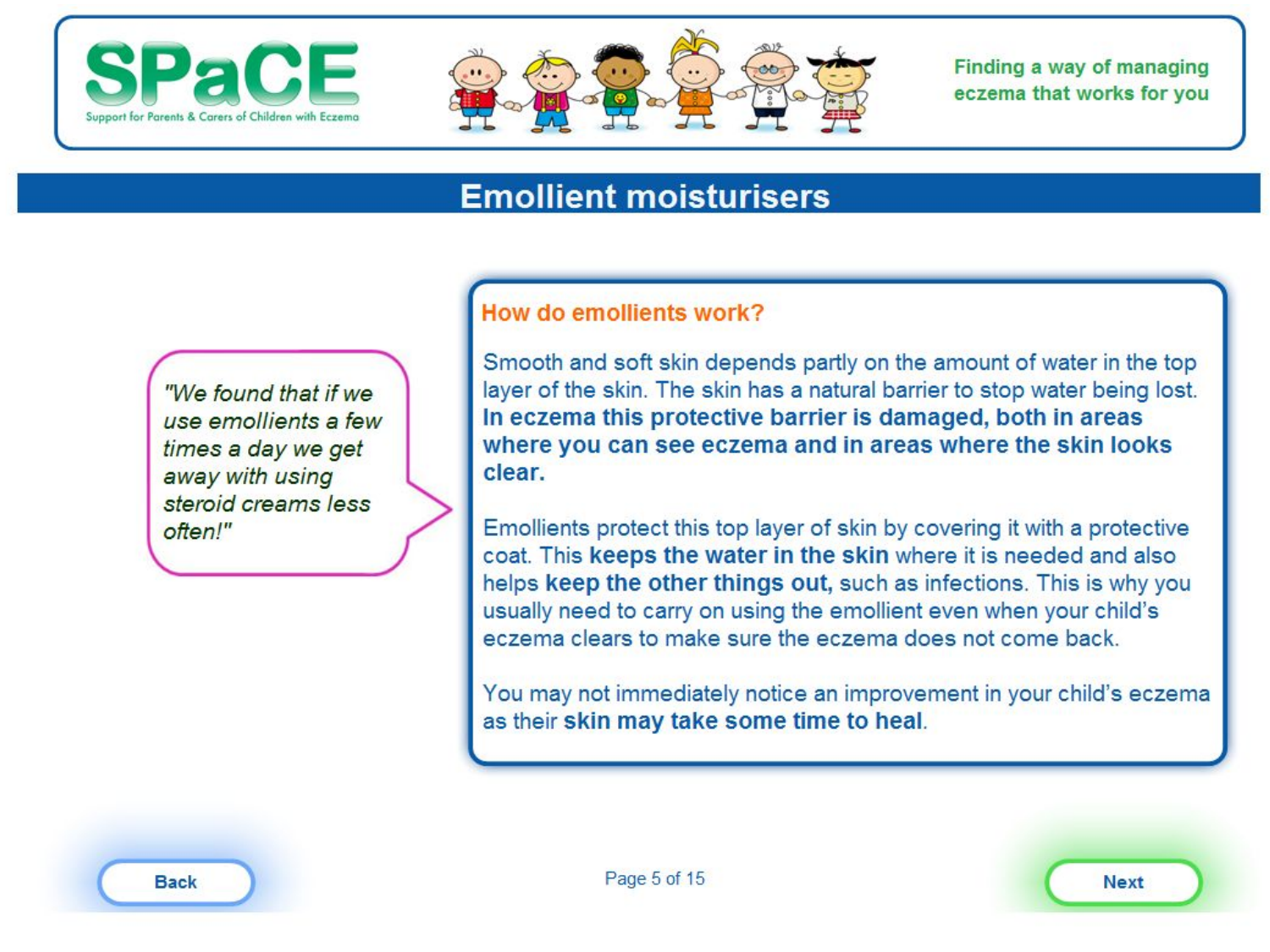

## Slide 11
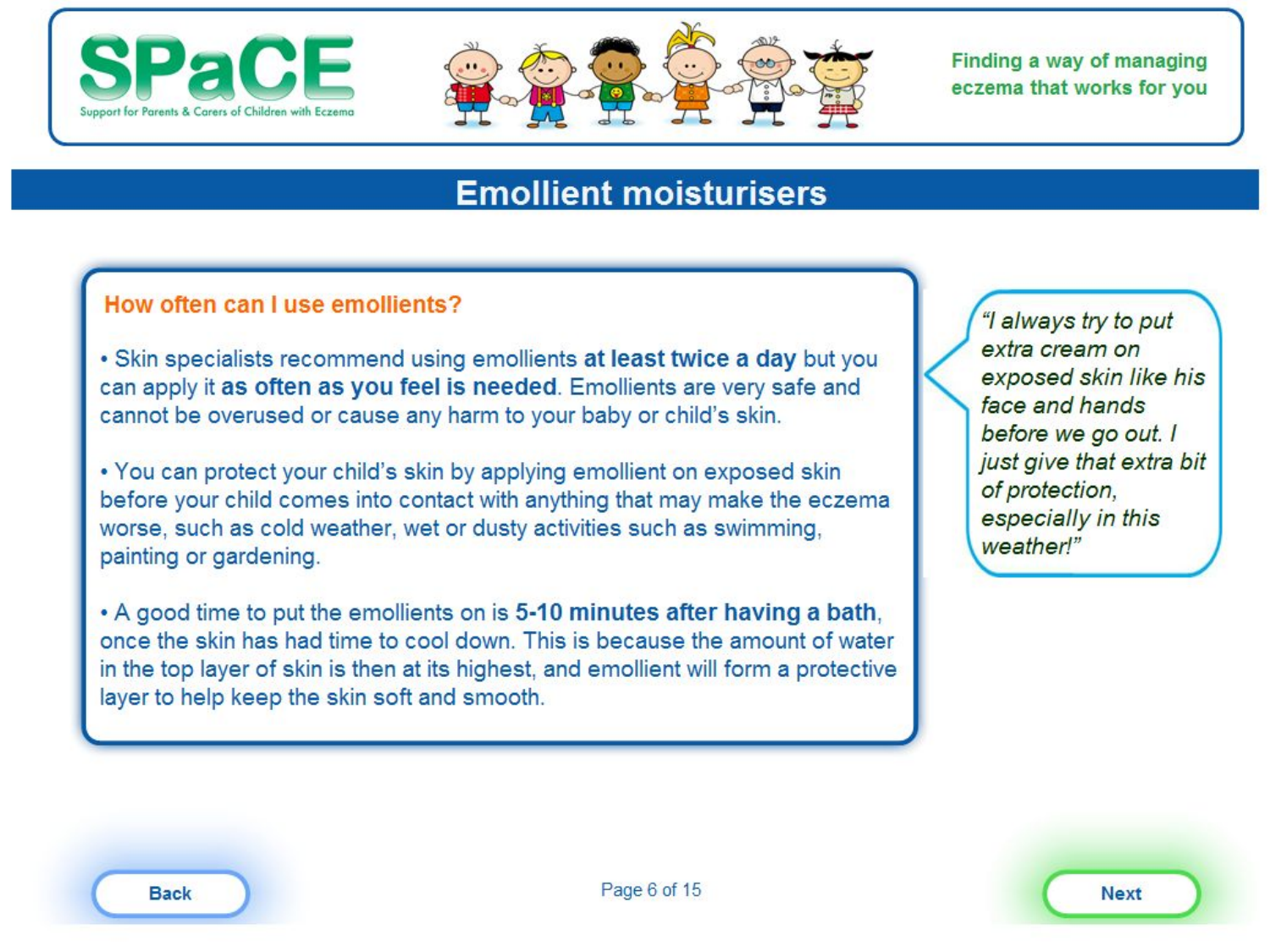

## Slide 12
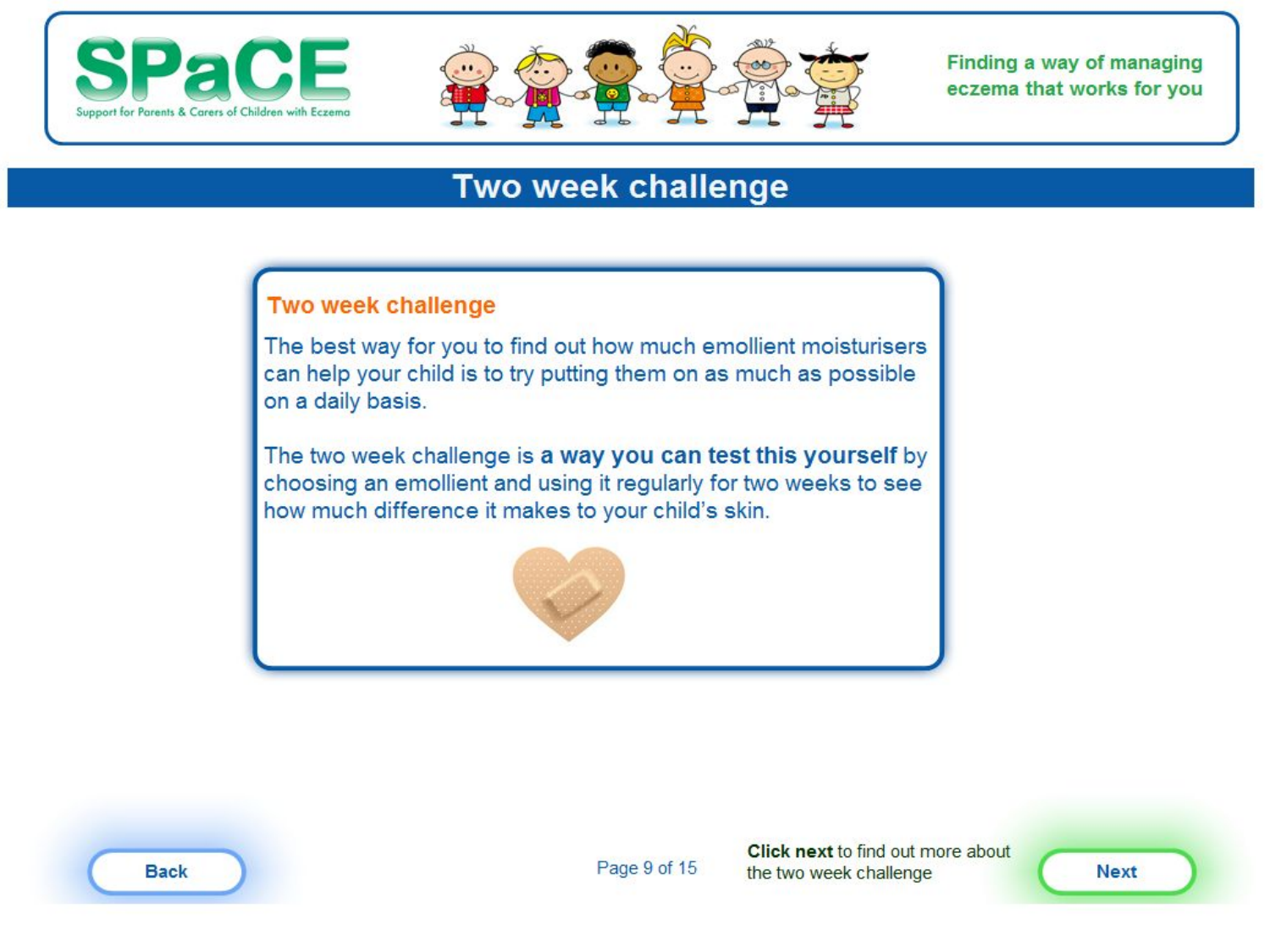

## Slide 13
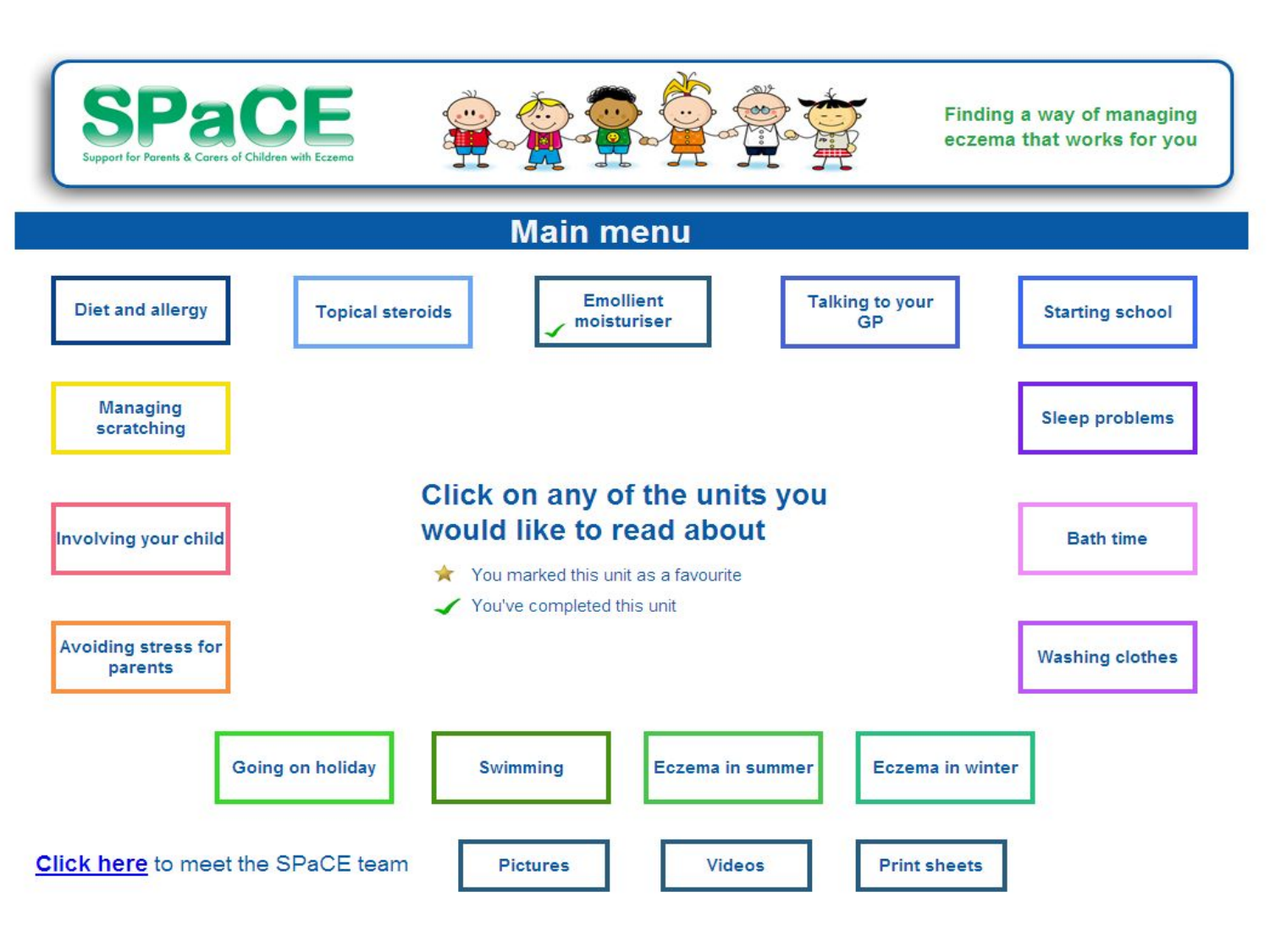

Supplement: Supplementary file 1 [file jmir_v16i3e70_app1.pptx]
